# Bucillamine Prevents Afatinib-Mediated Inhibition of Epidermal Growth Factor Receptor Signaling

**DOI:** 10.3390/ph12040165

**Published:** 2019-11-07

**Authors:** Naoyuki Nishiya, Moeka Murai, Ayumi Hosoda, Honami Yonezawa, Norikazu Omori

**Affiliations:** 1 Division of Integrated Information for Pharmaceutical Sciences, Department of Clinical Pharmacy, Iwate Medical University School of Pharmacy, 1-1-1 Idaidori, Yahaba-cho, Shiwa-gun, Iwate 028-3694, Japan; kalanchoe0321@icloud.com (M.M.); ayumayum999@gmail.com (A.H.); honamiy@iwate-med.ac.jp (H.Y.); skyline-fr@live.jp (N.O.); 2 Department of Pharmacy, Iwate Medical University Hospital, 2-1-1 Idaidori, Yahaba-cho, Shiwa-gun, Iwate 028-3695, Japan

**Keywords:** adverse effect, EGFR-TKI, skin rash

## Abstract

Molecular targeting therapies often cause characteristic adverse effects, such as skin rash during anti-epidermal growth factor receptor (EGFR) therapies, making treatment continuation difficult. In contrast, skin symptoms induced by EGFR inhibition are strongly correlated with the overall survival of the therapies. Therefore, controlling adverse effects not only facilitates treatment continuation but also increases clinical benefits. In this study, we proposed a novel strategy for reducing EGFR–tyrosine kinase inhibitor (TKI)-induced adverse effects in nontumorous organs by repositioning approved medicines using a zebrafish model. We developed a model system for evaluating chemical quenchers of afatinib, a clinically available irreversible EGFR-TKI, by scoring the inhibition of afatinib-induced hyperformation of lateral line neuromasts in zebrafish larvae. Bucillamine, an antirheumatic drug, was identified as an afatinib quencher in the zebrafish system and inhibited TKI activity in vitro. In addition, bucillamine restored EGFR autophosphorylation and downstream signaling in afatinib-treated A431 cells. Thus, topical bucillamine is a potential reliever of irreversible EGFR-TKI-induced skin rash. The zebrafish model can be applied to a screening for quenchers of other anti-EGFR-targeting therapies, including reversible TKIs and biologics.

## 1. Introduction

Abnormal activation of the epidermal growth factor receptor (EGFR) by mutation or overexpression leads to the development and progression of cancers in various organs [1]. Small molecule EGFR–tyrosine kinase inhibitors (EGFR-TKIs) and monoclonal antibodies against EGFR have shown strong efficacy in EGFR-dependent cancers [1]. Compared to classical chemotherapeutics which affects most proliferating cells, EGFR-targeting therapies decrease the incidence of systemic adverse effects. However, EGFR-TKIs and antibodies commonly cause characteristic cutaneous toxicities, such as papulopustular follicular rash, pruritus, skin xerosis, and nonscarring alopecia, in a high frequency [1,2,3], resulting in treatment interruption or discontinuation, which might interfere with treatment efficacy [4]. In contrast, skin rash severity is positively correlated with clinical response and predicts the efficacy of EGFR-targeting therapies [4,5]. Therefore, for better clinical outcomes, especially for potential high responders with severe symptoms, it is important to control the emergence and severity of skin rash during EGFR-targeting therapies. 

The correlation between skin rash severity and treatment efficacy suggests the existence of a common mechanism underlying skin rash and anticancer treatment [4,5]. Non-small cell lung cancer (NSCLC) patients treated with erlotinib, an EGFR-TKI, show altered differentiation of the pilosebaceous epithelium as the primary process of EGFR inhibition in the human skin and is hypothesized as a trigger of inflammation and skin rash [6]. Genetic ablations of *EGFR* in animal models show abnormalities in the differentiation of keratinocytes in hair follicles [7,8]. Pharmacological inhibition of EGFR or its downstream component, extracellular signal-regulated kinase 1/2 (ERK1/2), induce chemokine expression in keratinocytes and skin inflammation [9,10]. In addition, mouse models lacking epidermal EGFR expression have demonstrated that EGFR signaling in keratinocytes controls skin inflammation and innate immunity, providing an insight into the mechanisms underlying the adverse effects of EGFR inhibitors [11,12]. 

Topical restoration of EGFR signaling in the skin might mitigate skin rash in patients treated with EGFR-targeting therapies, without affecting anticancer activity in tumor lesions. A chemical compound that inhibits EGFR-TKI activity in the normal epidermis can be used to control adverse effects, leading to treatment continuation and increased clinical benefits. In this study, we proposed a novel strategy for identifying a chemical quencher of afatinib, a clinically available, irreversible EGFR-TKI possessing a reactive acrylamide group that causes frequent and severe cutaneous adverse events. First, we evaluated EGFR-TKI activity by exploiting extra lateral line neuromast development in zebrafish larvae as an in vivo model. Then, Food and Drug Administration (FDA)-approved medicines with sulfhydryl groups were assessed to determine whether they reduce the number of neuromasts increased by afatinib and whether a candidate inhibits EGFR-TKI activity in vitro and in human cells.

## 2. Results

### 2.1. Afatinib Induces Extra Formation of Lateral Line Neuromasts in Zebrafish Larvae

PD168393 is an irreversible pan ErbB (EGFR) family kinase inhibitor for research [13], and its action leads to the extra-neuromast phenotype in zebrafish larvae [14]. Therefore, we hypothesized that a clinically available EGFR-TKI also results in the extra-neuromast phenotype and that a quenching compound for an EGFR-TKI rescues the phenotype, so the EGFR-TKI quencher can be evaluated and ultimately screened by monitoring the phenotype. Adverse events affecting the skin caused by afatinib, an irreversible EGFR-TKI, are more frequent and severe compared to gefitinib, a reversible EGFR-TKI [15]. Therefore, we selected afatinib as an inducer of the extra-neuromast phenotype. 

**Figure 1:**
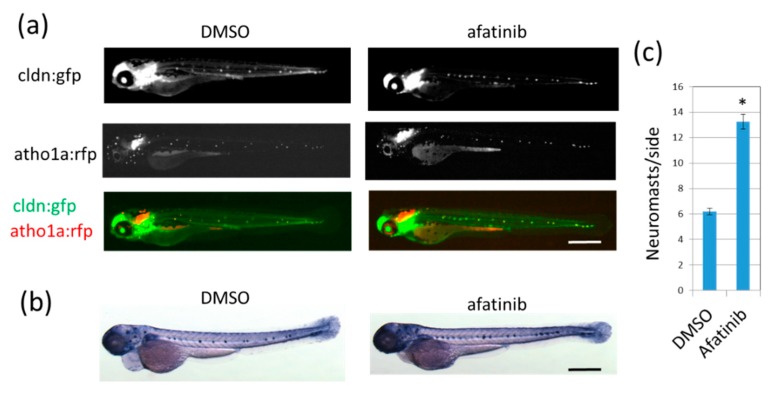
Afatinib induces the development of extra lateral line neuromasts in zebrafish larvae. (**a**,**b**) Neuromasts of 72 hpf larvae treated with DMSO (left) or afatinib (right) from 26 hpf were detected by fluorescence from *cldn*:*gfp*; *atoh1*:*rfp* transgenic larvae (**a**) or by endogenous ALP activity (**b**). (**a**) Fluorescence from *cldnb*:*gfp* highlighted neuromast cells and the epidermis, and fluorescence from *atoh1a*:*rfp* highlighted neuromast hair cells. GFP and RFP images were merged. Compared to DMSO treatment, increased neuromasts were observed in afatinib-treated zebrafish larvae. Scale bar = 500 µm. (**c**) The number of neuromasts on one side were counted. The graph indicates mean ± SEM (*n* = at least 54). * *p* < 0.01; hpf, hours postfertilization; DMSO, dimethyl sulfoxide; ALP, alkaline phosphatase; GFP, green fluorescent protein; RFP, red fluorescent protein; SEM, standard error of the mean.

We used the *cldn*:*gfp*; *atoh1*:*rfp* transgenic zebrafish line that expresses green fluorescent protein (GFP) in neuromast cells and the epidermis and red fluorescent protein (RFP) in neuromast hair cells [16]. Treatment of 26-hour-postfertilization (hpf) larvae with 10 μM afatinib induced the formation of the extra-neuromast phenotype, which is up to double that of DMSO-treated 72 hpf control larvae (5–8 neuromasts/side; Figure 1a). Differentiated neuromasts have high endogenous alkaline phosphatase (ALP) activity, so 72 hpf larvae were also stained with the nitro blue tetrazolium/5-bromo-4-chloro-3-indolyl phosphate (NBT/BCIP) reagent (Figure 1b,c). These data indicated that afatinib can induce the extra-neuromast phenotype. 

### 2.2. Bucillamine Is an Irreversible EGFR-TKI Quencher 

**Figure 2:**
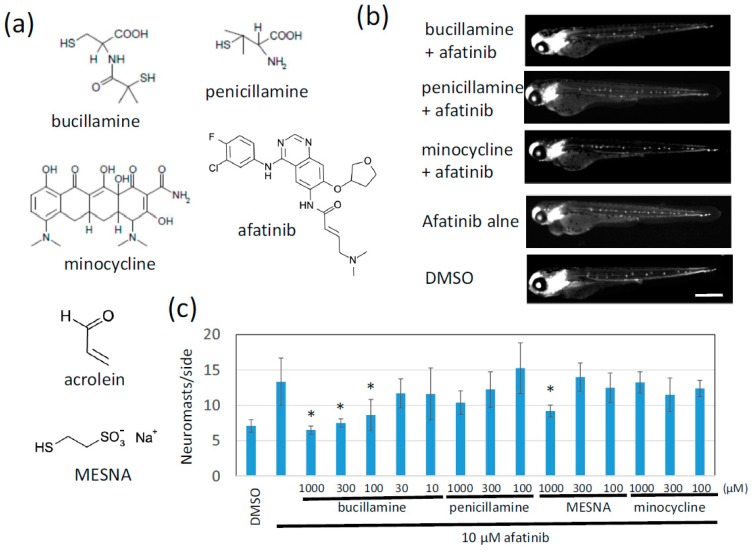
Bucillamine, an antirheumatic drug, suppressed afatinib-induced extra lateral line neuromast development. (**a**) Structures of the test compound, acrolein, and MESNA are shown. (**b**) Clinically available sulfhydryl compounds were evaluated for afatinib quenchers. Neuromasts of 72 hpf larvae treated with the indicated test compound were detected by fluorescence of *cldnb*:*gfp*. Scale bar = 500 µm. (**c**) The number of neuromasts on one side were counted. The graph indicates the mean ± SD (*n* = at least 4). * *p* < 0.01; MESNA, sodium 2-mercaptoethanesulfonate; hpf, hours postfertilization; SD, standard deviation.

Sodium 2-mercaptoethanesulfonate (MESNA) is a detoxicant used for hemorrhagic cystitis caused by alkylating anticancer drugs, such as cyclophosphamide and ifosphamide. These drugs are converted to an active metabolite and a byproduct acrylaldehyde (acrolein), which is chemically active and the main cause of hemorrhagic cystitis [17]. The MESNA sulfhydryl group traps and quenches acrolein through Michel addition before reacting with the bladder epithelium [18] (Figure 2a). By taking an analogy of the MESNA-mediated detoxification of acrolein, we hypothesized that a sulfhydryl compound forms conjugates with an acrylamide group of afatinib through Michel addition. 

The classical FDA-approved antirheumatic drugs penicillamine and bucillamine contain one or two reactive sulfhydryl groups (Figure 2a). We tested these two drugs in the zebrafish phenotypic evaluation system to determine whether they cancel the afatinib-induced extra-neuromast phenotype. Afatinib reproducibly induced the formation of extra neuromasts, twice as many as the control larvae. Bucillamine significantly decreased the number of neuromasts back to the level of control larvae in a dose-dependent manner (Figure 2b,c). Penicillamine and MESNA also tended to inhibit afatinib activity. Interestingly, minocycline, which is clinically used to relieve skin rash caused by EGFR-targeting therapies [19], had no effect or was much less effective compared to bucillamine (Figure 2b,c). 

### 2.3. Bucillamine Forms Adducts With Afatinib and Prevents In Vitro Kinase Inhibition of Afatinib 

**Figure 3:**
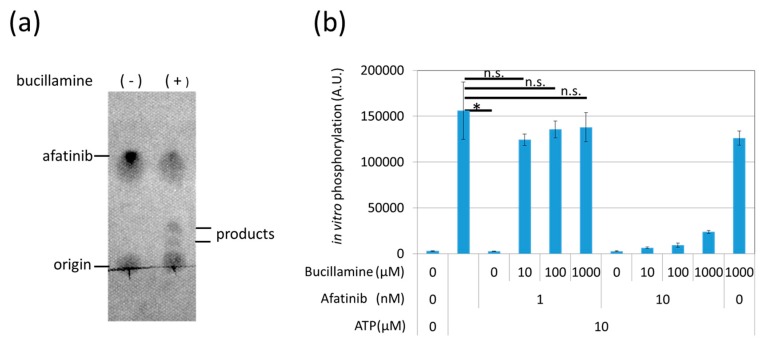
Bucillamine inhibited afatinib-mediated EGFR kinase inhibition in vitro. (**a**) 10 μM afatinib was incubated with DMSO or 1 mM bucillamine for 90 min at 25 °C. The reaction products were analyzed by TLC with UV detection. (**b**) A recombinant EGFR kinase domain was pre-incubated with 1 or 10 nM afatinib in the presence or absence of 10–1000 μM bucillamine. Then, 10 μM ATP was added to the kinase in order to initiate phosphorylation of an artificial substrate peptide. Phosphorylation levels were measured by ELISA using antiphosphotyrosine antibody. The graph indicates the mean ± SEM (*n* = at least 3). DMSO, dimethyl sulfoxide; EGFR, epidermal growth factor receptor; TLC, thin-layer chromatography; UV, ultraviolet; ATP, adenosine triphosphate; ELISA, enzyme-linked immunosorbent assay; SEM, standard error of the mean.

Phenotypic chemical screening has the potential to identify upstream and downstream signaling modulators [20], but it is often difficult to reach a direct target molecule. To confirm that bucillamine directly reacts with afatinib, bucillamine was incubated with afatinib in vitro, and the reaction products were separated by thin-layer chromatography (TLC); afatinib and the reaction products were detected by UV absorption. Afatinib alone showed a single spot after TLC, while two spots with different mobility compared to afatinib appeared in the reactant in the presence of bucillamine (Figure 3a), suggesting that bucillamine directly reacts with afatinib and forms reaction products, although the products have not yet been identified.

Next, to test whether bucillamine restores EGFR kinase activity in the presence of afatinib, we analyzed an in-vitro kinase reaction using a recombinant kinase domain of wild-type (WT) human EGFR. The EGFR kinase was activated by adding adenosine triphosphate (ATP) and phosphorylated a biotinylated artificial substrate peptide. Kinase activation was quantified by enzyme-linked immunosorbent assay (ELISA) with avidin-coated plates and an antiphosphotyrosine antibody. Results showed that 1 nM afatinib completely inhibits EGFR kinase activation (Figure 3b). However, the addition of bucillamine restored EGFR kinase activation to the no-inhibitor condition, even in the presence of 1 nM afatinib (Figure 3b). These findings indicate that bucillamine can suppress afatinib-mediated EGFR kinase inhibition. 

### 2.4. Bucillamine Restores EGFR Autophosphorylation and Its Downstream Signaling in Afatinib-Treated A431 Cells 

**Figure 4:**
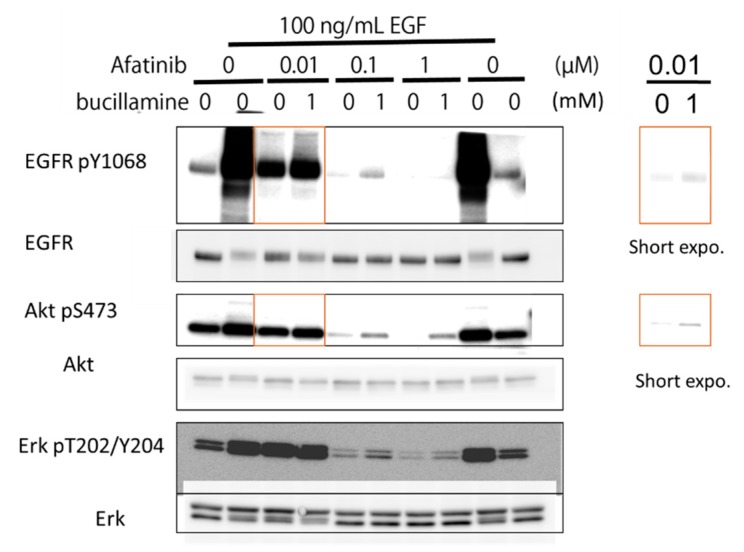
Bucillamine inhibited afatinib-mediated inhibition of EGFR autophosphorylation and downstream signaling. A431 cells expressing endogenous WT EGFR were pre-incubated with 0.01–1 μM afatinib in the presence or absence of 1 mM bucillamine. Then, 100 ng/mL of EGF was added to the culture medium. After 15 min incubation, cell lysates were prepared and analyzed by Western blotting with specific antibodies, as indicated. Shorter-exposure images are shown on the right. EGFR, epidermal growth factor receptor; WT, wild type.

To evaluate the effects of bucillamine on EGFR-signaling inhibition by an irreversible EGFR-TKI, the human epidermoid carcinoma A431 cell line was treated with afatinib with or without bucillamine. A431 cells were stimulated with 100 ng/mL of human EGF, increasing autophosphorylation of endogenous WT EGFR and activation of the downstream effectors protein kinase B (AKT) and ERK1/2 (Figure 4). Then, 1 mM bucillamine was added under various treatment conditions of A431 cells with afatinib. Results showed that the addition of bucillamine restored EGFR autophosphorylation and reactivated AKT and ERK1/2 partially, indicating that bucillamine attenuates afatinib-mediated inhibition of EGFR signaling in human cells. 

## 3. Discussion

Although EGFR-targeting therapies show remarkable efficacy, their characteristic cutaneous toxicities interrupt and discontinue treatment and interfere with clinical benefits [1,2,3,4]. Local prevention of EGFR inhibition in the skin might prevent or cure the skin rash caused by disruption of normal EGFR functions by the targeting therapeutics, without affecting anticancer activity, ensuring continuous treatment and, ultimately, clinical benefits. In this study, we proposed a novel strategy for identifying a chemical quencher to detoxify an irreversible EGFR-TKI. We developed an evaluation system for the chemical quencher using an extra-neuromast phenotype in zebrafish larvae with reduced EGFR family kinase activity. The evaluation system identified bucillamine, which is an antirheumatic drug possessing two sulfhydryl groups, as a chemical quencher against afatinib, an irreversible EGFR-TKI. Bucillamine restored EGFR kinase activation in vitro and EGFR signaling in human epidermoid carcinoma A431 cells. These findings suggested that bucillamine can be repositioned as a detoxicant against irreversible EGFR-TKIs. 

Extra neuromast formation in zebrafish larvae is an in vivo model that reflects the inhibition of pan-EGFR family kinases. Afatinib increased the number of lateral line neuromasts in zebrafish larvae (Figure 1). Large-scale genetic screening of zebrafish has classified mutants with abnormal phenotypes in inner ears and/or lateral lines, including *hypersensitive* mutant which has extra neuromasts [14,21]. Mapping a corresponding gene for the *hypersensitive* phenotype has identified one of the EGFR family genes, *HER3* (*ERBB3*) [14,21,22]. Pharmacological inhibition of pan-EGFR family kinases also leads to extra neuromast formation [14,23]. These findings indicate that the inhibition of pan-EGFR family kinases, including HER3, generally induces extra neuromast formation. Pharmacological inhibition of pan-EGFR family kinases might involve a risk of off-target effects but might provide the benefit that a quenching agent, which chemically reacts or causes drug–drug interaction, can be identified in a screening for inhibitors of an EGFR-TKI, such as bucillamine in this study. Therefore, the extra-neuromast phenotype caused by pharmacological pan-EGFR inhibition creates an in-vivo model that can be applied to a screening for EGFR-TKI quenchers that chemically inactivate TKIs, affect the pharmacokinetics of TKIs, or restore downstream signaling.

Bucillamine, an antirheumatic drug, inhibits the EGFR-TKI activity of afatinib through direct reaction. Bucillamine inhibits the afatinib-induced phenotype in a dose-dependent manner (Figure 2b,c). In addition to the in-vivo system, bucillamine and afatinib form reaction products with slower mobility in TLC compared to afatinib alone (Figure 3a). Bucillamine also diminishes afatinib-mediated inhibition of recombinant EGFR kinase activation in vitro (Figure 3b). In addition, bucillamine restores EGFR autophosphorylation and downstream signaling in afatinib-treated A431 cells (Figure 4). Although details of the antirheumatic activity of bucillamine are unknown, the structurally-related antirheumatic drug penicillamine dissociates S–S bonds in rheumatoid factors and the immune complex with its sulfhydryl residue [24]. Interestingly, compounds possessing a single sulfhydryl group (penicillamine and MESNA) showed a tendency to inhibit afatinib activity, but the inhibition was weaker than bucillamine, which has two sulfhydryl groups. This difference may be explained by doubled reactivity in bucillamine. On the other hand, a non-sulfhydryl compound, minocycline, did not rescue the exra neuromast phenotype (Figure 2). Minocycline has been used for EGFR inhibitor-induced dermatologic conditions because of its anti-inflammatory effects rather than antibiotic effects [25,26]. Therefore, our screening system may be suitable for searching a quencher that is directly reacting with an irreversible EGFR-TKIs, but not for a compound with a mechanism related to higher order biological processes, such as immune responses. We hypothesize that bucillamine can inactivate other irreversible EGFR-TKIs possessing an acrylaldehyde gorup, such as dacomitinib [27] and osimertinib [28]. Further screening of sulfhydryl compounds with similar activities to bucillamine might identify a more potent quencher than bucillamine. 

Topical administration of bucillamine to the skin should relieve the drug-induced cutaneous adverse effects of an irreversible EGFR-TKI without affecting its anticancer activity. Bucillamine has limited quenching activity; therefore, a substantial amount of bucillamine is needed in order to inactivate irreversible EGFR-TKIs (Figure 4). This may cause local dermatitis as a known adverse effect of bucillamine. Furthermore, topically administered bucillamine might be transdermally absorbed and distributed systemically. However, bucillamine diluted by blood and tissue fluid is not enough concentration to inactivate afatinib, resulting in an ideal adverse effect-reliever without interfering with anticancer activity. In contrast, systemic detoxification by bucillamine might be useful in cases of accidental overdosing of irreversible EGFR-TKIs. In addition, the anti-inflammation activity of bucillamine might help reduce adverse effects [29,30,31,32]. Future study is required for confirming the quenching activity of bucillamine in normal skin in a mammalian model. A large scale screening will identify compounds that restore downstream signaling as detoxicants for reversible TKIs and antibodies, such as gefitinib, erlotinib [3], cetuximab [3,33], and panitumumab [3,34].

## 4. Materials and Methods 

### 4.1. Reagents and Cell Lines

Bucillamine was purchased from FUJIFILM Wako Chemicals USA Corporation (Richmond, VA, USA) or Santa Cruz Biotechnology (Dallas, TX, USA); penicillamine, MESNA, and minocycline from Sigma; afatinib and afatinib dimaleate from Selleck (Houston, TX, USA. Antibodies against EGFR and pEGFR (Y1068) were purchased from Cell Signaling Technology (Beverly, MA, USA). Horseradish peroxidase (HRP)-conjugated anti-rabbit immunoglobulin G (IgG) and anti-mouse IgG antibodies were purchased from GE Healthcare (Chicago, IL, USA). The A431 cell line was obtained from the American Type Culture Collection (Manassas, VA, USA) and cultured in Roswell Park Memorial Institute (RPMI) 1640 medium supplemented with 2 mM L-glutamine and 10% fetal bovine serum in a humidified 5% CO_2_ atmosphere at 37 °C.

### 4.2. Zebrafish

The *cldn*:*gfp*; *atoh1*:*rfp* double-transgenic zebrafish line was obtained from Dr. Hironori Wada (Kitasato University) and maintained, as previously described [16]. *cldn*:*gfp*; *atoh1*:*rfp* was obtained by crossing *Tg*(*cldn:gfp*)*^zf106^* and *Tg Tg*(*atoh1:rfp*)*^nns8^* [16]. Zebrafish larvae were arrayed in round-bottomed 96-well plates and treated with test compounds at 26 hpf. Tested compounds were chosen by the following criterion: FDA-approved drugs, clinically available drugs in Japan, and modes of action related to reactive sulfhydryl groups. After 30–60 min incubation, the embryos were treated with 10 μM afatinib and incubated in a humidified box at 28.5 °C. At 72 hpf, the larvae were observed under a fluorescent microscope or an IN Cell Analyzer (GE Healthcare). Student’s t-test was used for statistical analysis. This study was approved by the animal care ethical committee of Iwate Medical University, Iwate, Japan (permit no. 30–023). Fish care and experimental procedures were conducted in accordance with the *Guide for Animal Experimentation* of the Animal Care Ethical Committee, Iwate Medical University.

### 4.3. Alkaline Phosphatase (ALP) Staining

For ALP staining, zebrafish larvae were fixed in 4% paraformaldehyde (PFA) for 1 h at room temperature. Then, the larvae were washed twice for 5 min each in phosphate-buffered saline (PBS)/0.1% Tween-20 and then washed again twice for 15 min each in staining buffer (50 mM MgCl_2_, 100 mM NaCl, 100 mM Tris-HCl [pH 9.5], and 0.1% Tween-20). The larvae were then placed in staining buffer plus NBT/BCIP (Roche Holding AG, USA) and stained in the dark at room temperature. The staining reaction was stopped with 4% PFA.

### 4.4. Thin-layer Chromatography (TLC)

In a reaction buffer (120 mM 4-(2-hydroxyethyl)-1-piperazineethanesulfonic acid [HEPES] [pH 7.5], 10 mM MgCl_2_, 10 mM MnCl_2_), 0.2 mM Afatinib was incubated with or without 20 mM bucillamine for 90 min at 25 °C. Reaction products were spotted onto Chromato Sheet (Fujifilm Wako, Osaka, Japan) and developed in chloroform/methanol (8:1, vol/vol) solvent system. Each spot on the chromatogram was detected under a UV illuminator at 254 nm.

### 4.5. In Vitro Kinase Assay

We pre-incubated 100 ng of recombinant cytoplasmic domains (amino acid residues 696 to the C-terminus) of the WT EGFR (Carna Bioscience, Kobe, Hyogo, Japan) with kinase inhibitors in 25 μL of kinase reaction buffer (120 mM 4-(2-hydroxyethyl)-1-piperazineethanesulfonic acid [HEPES] [pH 7.5], 10 mM MgCl_2_, 10 mM MnCl_2_, 6 μM Na_3_VO_4_, and 2.5 mM dithiothreitol (DTT]) for 30 min at 25 °C. Subsequently, 25 μL of the ATP/substrate solution containing 20 μM ATP and 6 μM poly-(Glu-Tyr)-biotinylated peptide (Cell Signaling Technology, Danvers, MA, USA) was added to the pre-incubation mixture. The kinase reaction was performed for 30 min at 25 °C and terminated by adding 50 μL of stop buffer (50 mM ethylenediaminetetraacetic acid [EDTA], pH 8.0). Kinase activity was estimated using ELISA with avidin-coated 96-well plates and antiphosphotyrosine antibodies (PY20).

### 4.6. Phosphorylation Analysis

A431 cells were pretreated with bucillamine for 15 min and with afatinib for 60 min in 0.2% serum conditions and stimulated with 200 ng/mL of EGF for 15 min. Alternatively, NCI-H1975 cells were treated with kinase inhibitors at the indicated concentration for 16 h. Next, the cells were lysed in radioimmunoprecipitation assay (RIPA) buffer (50 mM Tris-HCl [pH7.4], 150 mM NaCl, 1% Triton X-100, 1% sodium deoxycholate, 0.1% sodium dodecyl sulfate [SDS], 1 mM NaF, 1 mM Na_3_VO_4_, and protease inhibitors) and cleared by centrifugation. SDS sample buffer was added to the supernatants, and protein samples were separated by sodium dodecyl sulfate–polyacrylamide gel electrophoresis (SDS-PAGE) and analyzed using Western blotting with antibodies against phospho-specific or total proteins.
